# Effect of Acupuncture on Premature Ovarian Failure: A Pilot Study

**DOI:** 10.1155/2014/718675

**Published:** 2014-03-10

**Authors:** Yingru Chen, Yigong Fang, Jinsheng Yang, Fei Wang, Yingying Wang, Li Yang

**Affiliations:** ^1^Institute of Acupuncture and Moxibustion of China Academy of Chinese Medical Sciences, No. 16 Dong Zhi Men Nei Nan Xiao Jie, Dongcheng District, Beijing 100700, China; ^2^Institute of Acupuncture and Moxibustion of the Hospital Affiliated to Tianjin University of Traditional Chinese Medicine, No. 314 An Shan Xi Dao, Nankai District, Tianjin, 300193, China

## Abstract

To investigate the effect of acupuncture on women with premature ovarian failure (POF), prospective consecutive case series study was applied. 31 women with POF were included; all patients were treated with acupuncture once every other day, three times a week for three months. Acupoints, GV 20, GV 24, GB 13, CV 3, CV 4, BL 23, BL 32, ST 25, ST 28, ST 29, ST 36, SP 6, KI 3, and LR 3, were selected. Serums FSH, E_2_, and LH level, Self-Rating Anxiety Scale (SAS), and Kupperman score were measured at baseline and at the end of treatment; the menstrual cycle was recorded during one-month follow-up. Compared with baseline, patients' serums FSH and LH were decreased, E_2_ was increased, and SAS score and Kupperman score were decreased. Four patients resumed menstrual cycle after treatment and two resumed during follow-up. No serious adverse events were found in all patients. The results indicate that acupuncture may decrease serums FSH and LH level, raise serum E_2_ level, relieve anxiety, reduce mental stress, and improve the menopausal symptoms.

## 1. Introduction

Premature ovarian failure (POF) is a common cause of infertility in women and is characterized by amenorrhea before the age of 40. Menopause before the age of 40 is considered to be premature and is nonphysiological disorder. POF, also known as premature menopause or premature ovarian insufficiency, is defined by the presence of menopausal-level serum follicle-stimulating hormone (FSH) in women younger than 40 years [1–3]; the mean (±SD) age of natural menopause is 50 ± 4 years [4]. This syndrome is associated with the symptoms and metabolic effects of sex steroid deficiency, as well as the emotional sequelae experienced by couples who have difficulty in conceiving a pregnancy. POF is a disorder affecting approximately 1% of women <40 years, 1/1,000 women by the age of 30, and 1/10,000 women by the age of 20.

Women with POF have been reported to have diminished general and sexual well-being, are less satisfied with their sexual lives, have increased risk for low bone density, earlier onset osteoporosis and fractures, impaired endothelial function, earlier onset of coronary heart disease, and increased cardiovascular mortality and total mortality, and have more anxiety, depression, somatization, sensitivity, hostility, and psychological distress than normal women [5–7].

POF was not nominally recorded in Traditional Chinese Medicine (TCM). However, according to TCM theory, POF can be pertained to amenorrhea according to the clinical manifestations. As early as in 1237 A.D., the first book about gynaecology and obstetrics of Chinese Medicine,* The Complete Book of Effective Prescriptions for Diseases of Women*, said that acupuncture and Chinese Herbal Medicine usually led to satisfying symptom relieving effects in treating some gynaecology disorders, such as endometriosis, infertility, amenorrhea, and menopausal syndrome [8]. In recent studies, acupuncture has been found effective in reducing hot flashes of bilaterally ovariectomized patients [9] and women undergoing perimenopause and menopause [10–12] and significantly improving serum estradiol (E_2_) level and Kupperman scores of perimenopause patients [13]. Acupuncture also has been proved to be effective in improving pregnancy outcomes in women undergoing* in vitro* fertilization (IVF) from summarized data of several previously published meta-analysis and lots of clinical trials [14, 15]. Although the therapeutic mechanisms of acupuncture in the above-mentioned disorders are not yet to be fully understood, a plausible hypothesis is that acupuncture may influence related hormone levels.

However, there is less evidence on the effectiveness of acupuncture for patients with POF. This study, therefore, was designed to assess the feasibility and safety of acupuncture for POF patients on the basis of TCM theory, which includes local and distal acupoints to reinforce liver and kidney, to regulate Qi and Blood, and to adjust mental activity.

## 2. Materials and Methods

### 2.1. Study Design

This was a prospective consecutive case series study performed at the Hospital of Acupuncture and Moxibustion of China Academy of Chinese Medical Sciences. Participants received 3 sessions of acupuncture treatment, delivered 3 times a week for 3 months. The data from patients include the serums FSH, E_2_, and luteinizing hormone (LH) level, Self-Rating Anxiety Scale (SAS) [16], and Kupperman score [17], (The SAS and Kupperman score have been validated and widely used in Chinese population [18, 19].) The menstrual cycles were recorded during one-month follow-up. Adverse events were tracked for 3 months from initial acceptance of acupuncture treatment to the end of treatment.

**Figure 1:**
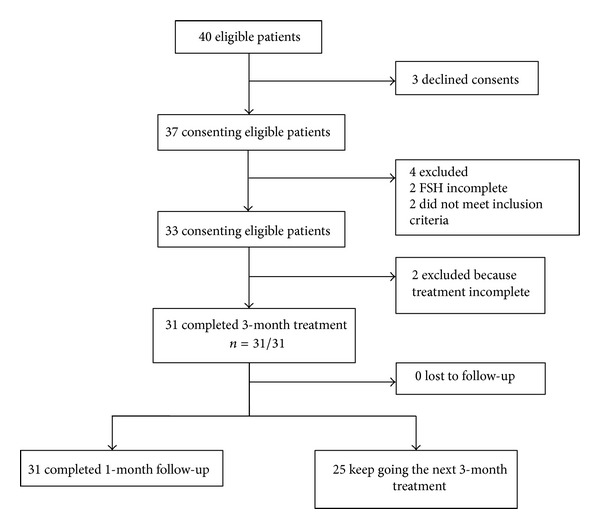
Flow chart of study.

The institute ethics committee approved this treatment protocol for women with POF (Institute of Acupuncture and Moxibustion Beijing (ethics) approval number: 20111117), and all patients signed informed consent before study participation. Acupuncture was performed by one therapist with more than 20 years of experience and Chinese medicine practitioner license from the Ministry of Health of the People's Republic of China. Data coordination, monitoring, analysis, and source verification were done by the researchers who did not take part in the clinical treatment.  Figure 1 shows the study design.

### 2.2. Study Participants

Present study recruited patients through the following ways: (1) publish recruiting advertisement on the website of the Hospital of Acupuncture and Moxibustion of China Academy of Chinese Medical Sciences; (2) publish recruiting notification on the largest Chinese doctor information website; (3) place recruiting advertisement in registration hall of our hospital. Informed consent was obtained before participant enrollment according to a clinical trial protocol. Recruitment was performed from December 2011 to January 2013, and 31 cases of POF were recruited.

Inclusion criteria were as follows: (1) the patients meet the POF diagnosis criteria, mainly with amenorrhea for four months or longer and FSH above 40 IU/L as detected on at least two occasions with at least one month apart [20–22]; (2) the patients' age is from 18 to 40; (3) before treatment, all patients had gone through one-month baseline evaluation period during which they stopped all medications influencing reproductive hormones; (4) patients were advised and agreed not to use these medications during study. Exclusion criteria were as follows: (1) bilateral oophorectomy, gonadal dysgenesis, and chromosomal abnormalities; (2) ovarian failure caused by radiotherapy and/or chemotherapy of cancer; (3) autoimmune diseases and/or receiving hormones or immunosuppressant drugs; (4) serious primary diseases of cardiovascular, liver, kidney, and hematopoietic system; (5) having no desire to participate in the research.

### 2.3. Interventions

*Acupuncture Needles*. Sterile, silver-handle, prepacked needles (HanYi single-use acupuncture needle, made in Tianjin HuaHong Medical Co., Ltd.) without guide tubes (size 0.25 mm × 25 mm, 0.25 mm × 40 mm, and 0.30 mm × 75 mm).

All acupoints were selected and localized according to WHO Standardized Acupuncture Point Location [23] (Table 1). Needles of the size of 0.25 mm × 25 mm were inserted horizontally into GV 20 (Baihui), GV 24 (Shenting), and the bilateral GB 13 (Benshen) with a depth of 20 mm and inserted perpendicularly into the bilateral KI 3 (Taixi) and LR 3 (Taichong) with a depth of 20 mm. 0.25 mm × 40 mm size needles were inserted perpendicularly into CV 3 (Zhongji), CV 4 (Guanyuan), the bilateral ST 25 (Tianshu), ST 28 (Shuidao), ST 29 (Guilai), ST 36 (Zusanli), and SP 6 (Sanyinjiao) with a depth of 30–35 mm. 0.30 mm × 75 mm size needles were inserted obliquely into the bilateral BL 32 (Ciliao, second sacral foramina) with a depth of 50–60 mm.

For acupuncture at BL 32 (Ciliao, second sacral foramina), patients should have a strong soreness sensation which radiates to the lower abdomen. For the other points, mild reinforcing-reducing method was used, and all patients should have the “De Qi” sensation (in which patients experience a radiating feeling considered to be indicative of effective needling).

The needles were administered for a maximum of 20–30 minutes, and acupuncture treatment was administered once every other day (two prescriptions were used alternatively, prescription 1 twice a week and prescription 2 once a week) for three months.

### 2.4. Study Outcomes

The data collected including the serums FSH, E_2_, and LH level, SAS, and Kupperman score, at baseline and after 3-month treatment. Additionally, patients' menstrual cycles were recorded according to individuals' reports during one-month follow-up. Adverse events were tracked for 3 months from initial acceptance of acupuncture treatment to the end of treatment.

### 2.5. Statistical Analysis

All analyses were done with SPSS software package (Version 17.0) using before and after measurements. Baseline characteristics of the patients were analyzed with conventional group descriptive statistics. *t*-test was used firstly for serums FSH, E_2_, and LH level, SAS, and Kupperman score. For the measurement data fitting normal distribution, using Two-Related-Samples Tests. If the measurement data did not fit the normal distribution, using Two-Related-Samples Tests Wilcoxon.

## 3. Results

### 3.1. Participant Flow

From December 2011 to May 2013, a total of 40 patients with POF visited the Hospital of Acupuncture and Moxibustion of China Academy of Chinese Medical Sciences seeking for acupuncture treatment. Of these patients, nine were excluded from the study with the following reasons: three patients declined to participate; two had incomplete FSH data; two did not meet inclusion criteria; two did not complete the whole session of treatment. (One patient moved to another city for job reason. The other one could not guarantee three times treatment per week after three-week treatment, because of her difficulties in asking for leave (Figure 1 and Table 2).)

Of these 31 patients, 25 had not been treated by acupuncture in the past, and six had been treated by acupuncture at least one month ago. And in the six, there were four received acupuncture treatment for cervical spondylosis, and two received acupuncture treatment for menstrual problem.

### 3.2. Baseline Characteristics

The baseline characteristics of the participants are shown in Table 2. The mean age at baseline was 35 years (SD = 4; range, 24~40). The mean history of amenorrhea was 8 months (SD = 6; range, 4~25). 26 of the 31 participants were with fertility request. No participant reported the use of hormone therapy or Chinese herbal therapy at baseline or during the entire observation period.

### 3.3. Effects of Acupuncture on Serums FSH and LH Level

**Figure 2:**
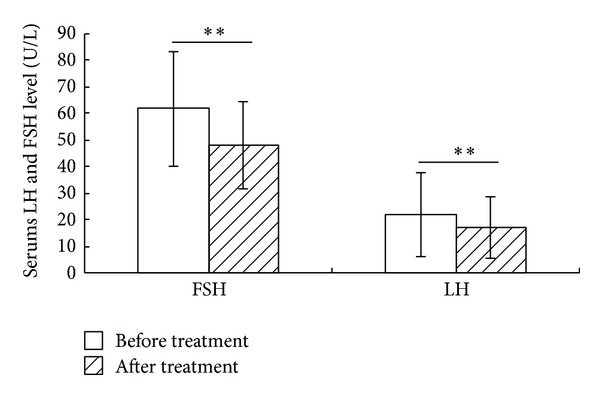
The serums LH and FSH level before and after acupuncture treatment. Both the serums FSH and LH level were reduced significantly after acupuncture treatment for 3 months (*P* = 0.001, *P* = 0.001, resp., *n* = 31) (**P < 0.01).

Changes in mean serum FSH level from baseline to 3 months after final acupuncture treatment session are presented in Figure 2. Two-Related-Samples Tests Wilcoxon showed a significant reduction in the average serum FSH level at the end of treatment (*Z* = 4.68, *P* = 0.001).

Changes in mean serum LH level from baseline to 3 months after the last time acupuncture treatment session are presented in Figure 2. Two-Related-Samples Tests showed a significant decrease in the average serum LH level at the end of treatment (*t* = 5.519, *P* = 0.001).

### 3.4. Effects of Acupuncture on Serum E_2_ Level

**Figure 3:**
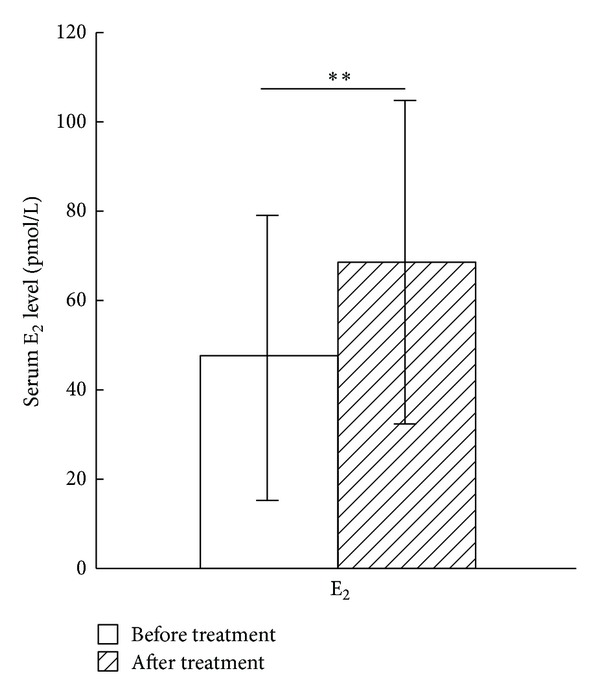
The serum E_2_ level before and after acupuncture treatment. The serum E_2_ level was reduced significantly after acupuncture treatment for 3 months (*P* = 0.001, *n* = 31) (**P < 0.01).

Changes in mean serum E_2_ level from baseline to 3 months after final acupuncture session are presented in Figure 3. Two-Related-Samples Tests Wilcoxon showed a significant reduction in the average serum E_2_ level at the end of treatment (*Z* = 4.48, *P* = 0.001).

### 3.5. Effects of Acupuncture on the Anxiety State Assessed by SAS and the Perimenopausal Syndrome via Kupperman Score

**Figure 4:**
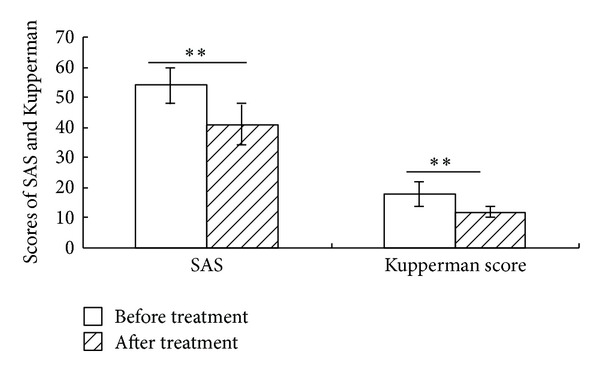
The scores of SAS and Kupperman before and after acupuncture treatment. Both the SAS and Kupperman scores were reduced significantly after acupuncture treatment for 3 months (*P* = 0.000, *P* = 0.000, resp., *n* = 31) (**P < 0.01).

Figure 4 showed that, during one-month baseline evaluation period, patients' SAS score was 54 ± 6. Two-Related-Samples Tests Wilcoxon showed a significant reduction to 41 ± 7 after 3-month acupuncture treatment (*Z* = 4.82, *P* = 0.000).

Figure 4 showed that, during one-month baseline evaluation period, patients' Kupperman score was 18 ± 4. Two-Related-Samples Tests Wilcoxon showed a significant reduction to 12 ± 2 after 3-month acupuncture treatment (*Z* = 4.71, *P* = 0.000).

### 3.6. Effects of Acupuncture on Menstrual Cycle

After 3-month treatment, six patients had improvement on menstruation (6/31, 19.4%). Four of them had menstruation after treatment, and two of them experienced menstruation during the one-month follow-up. Of the 6 patients who regained menstruation, 2 patients regained normal color, duration, and volume of period, and 4 patients had decreased menstrual flow but with normal color and duration as compared with normal menstrual bleeding.

### 3.7. Safety

During the 3-month treatment, two adverse events were reported by 2 patients (1 small haematoma and 1 needling pain after treatment). No serious adverse events were documented.

## 4. Discussion

Although POF was not nominally recorded in TCM, its clinical manifestations can be classified into amenorrhea according to TCM theory. The TCM etiology of amenorrhea includes insufficient transformation of blood of the spleen and stomach, severe consumption of yin blood and exhaustion of blood source, and blockage of blood stasis in the meridians and vessels due to retention of pathogenic factors in the uterus. In this study, local and distal acupoints with functions of reinforcing liver and kidney, regulating Qi and blood, and adjusting mental activity were selected according to the amenorrhea etiology of TCM theory.

The present results indicate that the acupuncture treatment can adjust patients' serums FSH, E_2_, and LH level and improve patients' SAS score and Kupperman score. The serum FSH level dropped to  48 ± 16.6 IU/L from baseline to the end of treatment (*P* = 0.001), the serum E_2_ level rose to  68.24 ± 36.15 pmol/L (*P* = 0.001), and the serum LH level dropped to  17.01 ± 11.66 (*P* = 0.001). The changes of hormones produced by acupuncture in present study were similar to the previous investigation using EA [24]. Modulation of serums FSH, E_2_, and LH level may partially explain the effects of acupuncture in treating POF, which is also observed in acupuncture improving other gynecological disorders [25].

Four patients regained menstrual cycle after treatment and two during one-month follow-up period of this study. Such result of present study is similar to the gaining of menstruation in a top athlete reported in a Japanese acupuncture study [26].

Patients' psychological comorbidities, signs, and symptoms were objectively measured with standard questionnaires by SAS and Kupperman score. The SAS score decreased to 41 ± 7 (*P* = 0.000 as compared with baseline), and Kupperman score decreased to 12 ± 2 (*P* = 0.000 as compared with baseline). The change of SAS and Kupperman score indicated that acupuncture may relieve anxiety, reduce mental stress, and improve menopausal symptoms (including hot flashes, night sweats, vaginal dryness, and mood swings).

Although POF is most frequently idiopathic or caused by autoimmune disorders, genetic causes, infections or inflammatory conditions, enzyme deficiencies, or metabolic syndromes [27], it also related to high mental and psychological distress [7]. Such cooccurrence of physical and psychological illnesses is not only associated with poor treatment response, lower quality of life, and increased healthcare costs but also related to the mutual influence of physiological processes and psychological distress that contribute to the development of POF. Present study just revealed that the SAS and Kupperman score of patients were lowered after three months' treatment. This might be explained by the mind adjustment and mental improving effects of acupuncture [28, 29].

Nonetheless, this present study only included 31 patients; thus the result of the study may not well characterize the general response of women with POF receiving acupuncture treatments. With an open label prospective study design and no control group, researcher could not eliminate these confounding factors. Of the 31 patients, there were two patients had acupuncture treatment in other hospitals 1.5 and 2 months before participating in our study. Because the detailed information of their previous acupuncture treatment (like acupoints selected and stimulating methods applied) was not fully recorded, we could not compare those two acupuncture regimens. This is a methodological drawback of present study. Thus, the detailed relevant information about previous treatment of participants should be fully and meticulously recorded in future study. Meanwhile, in order to increase the objectivity and fairness of observational study, inclusion and exclusion criteria of future study should be stricter. Excluding those patients who have had other treatments may reduce confounding factors.

Commonly used acupuncture formulas for amenorrhea in China normally include acupoints with functions of reinforcing liver and kidney and regulating Qi and blood. Compared to the commonly used formulas, our acupuncture regimen added acupoints for adjusting mental activity. This may partially explain the different therapeutic effects between the two patients' previous formula and ours.

Although the significant changes of serums FSH, E_2_, and LH level in this study were most likely due to acupuncture effects, those serum hormones' levels did still not reach the normal level after treatment. To test the therapeutic effectiveness of acupuncture, further randomized control trials are needed.

## 5. Conclusion

The present results showed the feasibility and safety of acupuncture for the treatment of POF in China. These findings suggest that acupuncture may decrease serums FSH and LH level, raise serum E_2_ level, relieve anxiety, reduce mental stress, and improve the menopausal symptoms. No serious side effects were found.

## Figures and Tables

**Table 1:** Acupoints used in this study.

Prescription	Acupoints
Prescription 1	CV 3 (Zhongji), CV 4 (Guanyuan), ST 25 (Tianshu), ST 28 (Shuidao), ST 29 (Guilai), and LR 3 (Taichong)
	GV 20 (Baihui), GV 24 (Shenting), GB 13 (Benshen), ST 36 (Zusanli), SP 6 (Sanyinjiao), and KI 3 (Taixi)

Prescription 2	BL 23 (Shenshu), BL 32 (Ciliao)
	GV 20 (Baihui), GV 24 (Shenting), GB 13 (Benshen), ST 36 (Zusanli), SP 6 (Sanyinjiao), and KI 3 (Taixi)

**Table 2:** Demographic and clinical characteristics at baseline.

Variable	*N* (%) or mean ± SD
Mean age, age	35 ± 4
Mean weight, kilogram	53.7 ± 7
Mean age at menarche, age	14 ± 1
History of amenorrhea, month	8 ± 6
History of pregnancy, *n*	15 (48)
History of abortion, *n*	13 (42)
History of delivery, *n*	5 (16)
Fertility request, *n*	26 (84)
